# A Microsimulation Model of Mpox in Los Angeles County: Implications for Future Disease Prevention and Control Strategies among Men Who Have Sex with Men

**DOI:** 10.1093/ofid/ofae401

**Published:** 2024-07-17

**Authors:** Citina Liang, Sze-chuan Suen, Chenglin Hong, Andrea Kim, Rita Singhal, Paul Simon, Mario Perez, Ian W Holloway

**Affiliations:** Daniel J. Epstein Department of Industrial and Systems Engineering, University of Southern California Viterbi School of Engineering, Los Angeles, California, USA; Daniel J. Epstein Department of Industrial and Systems Engineering, University of Southern California Viterbi School of Engineering, Los Angeles, California, USA; School of Social Work, University of Connecticut, Hartford, Connecticut, USA; Los Angeles County Department of Public Health, Los Angeles, California, USA; Los Angeles County Department of Public Health, Los Angeles, California, USA; Department of Epidemiology, University of California Los Angeles Fielding School of Public Health, Los Angeles, California, USA; Los Angeles County Department of Public Health, Los Angeles, California, USA; Department of Social Welfare, Luskin School of Public Affairs, University of California Los Angeles, Los Angeles, California, USA

**Keywords:** Los Angeles, men who have sex with men (MSM), microsimulation, mpox, people with HIV (PWH)

## Abstract

**Background:**

The 2022 monkeypox (mpox) outbreak in Los Angeles County (LAC) emphasized the need to prepare for emergent infectious disease outbreaks. Vaccination and promotion of sexual risk reduction practices appeared successful in LAC, as mpox cases declined starting in August. Nonetheless, questions persisted regarding the effectiveness of targeting vaccinations and the role of sexual risk reduction in reducing mpox cases.

**Methods:**

We collaborated with the LAC Department of Public Health to develop a microsimulation for men who have sex with men (MSM). This model tracked mpox dynamics by age, race/ethnicity, and HIV status and was calibrated and validated against surveillance data. We simulated counterfactual scenarios to understand the effects of variation in vaccination rates, timing of vaccination rollout, vaccine allocation, and sexual contact rates.

**Results:**

In the simulation, doubling the vaccination rate reduced cumulative cases over a 40-week time horizon by 13% but would necessitate 88 995 additional doses. Initiating vaccination 2 weeks earlier decreased cases by 11%, while an 8-week delay yielded a 20% increase in cases. A 3-week earlier decrease in sexual contact rates reduced cumulative cases by 60%, while a 3-week delay resulted in a 95% increase. Prioritizing people with HIV (PWH) for vaccination reduced cumulative cases, while allocating vaccines to a single racial/ethnic group was not effective.

**Conclusions:**

Our study highlights the significance of policies to support timely vaccination and sexual partnership reduction to address mpox outbreaks among MSM. These findings also underscore the need to target vulnerable risk groups, such as PWH.

Europe and North America experienced an mpox (monkeypox) surge starting in May 2022. California led the United States in mpox cases, with Los Angeles County (LAC) reporting the highest numbers within the state [1, 2]. By December 9, 2022, LAC reported 2245 confirmed mpox cases [3]. Most mpox cases were among gay, bisexual, and other men who have sex with men (MSM), and people with HIV (PWH) were disproportionately impacted [4–9]. According to data from the Los Angeles County Department of Public Health (LACDPH), >45% of cases in LAC were among PWH.

The virus showed a high rate of human-to-human transmission during the summer of 2022, especially through sexual contacts [6, 8–10]. However, reported cases in LAC and in the United States indicated a significant decline, especially after August [1, 3]. In LAC, the 7-day average number of cases dropped to <2 by December 2022, after peaking at 39 in August 2022 [3]. There were several events that may have played a role in the decline of mpox cases. Starting July 2022, the LACDPH recommended and distributed JYNNEOS [11], a 2-dose vaccine developed against smallpox infection with demonstrated protection for mpox [12]. This followed the World Health Organization’s (WHO's) declaration in July of the mpox outbreak as a public health emergency of international concern [13]. In August 2022, the Centers for Disease Control and Prevention (CDC) also released guidelines for mpox management, suggesting isolation from infected individuals and reducing one-time sexual partnerships [14]. These interventions, from vaccination to public health guidance about sexual risk reduction, likely enhanced public awareness of mpox and reduced sexual behavior, a conclusion that is supported by prior studies [15–18]. The rapid decrease in incidence rates during this period suggested that control measures were effective, and prior literature suggests that vaccines, behavior change, and postinfection immunity may play a role in reducing mpox cases.

However, it is unclear to what extent vaccination efforts and sexual risk reduction messaging contributed to the decrease. Additionally, at the time, there was concern that sexual behavior that placed individuals at risk for mpox could increase again if the mpox threat was prematurely perceived to be over, potentially hindering disease control efforts [19]. Our study therefore attempted to quantify the contributions of these control efforts in LAC, particularly in light of uncertainty regarding transmission behavior. We focus on LAC as it represents an important metropolitan area at the epicenter of the mpox outbreak and has a diverse population of MSM. Our study is distinct in its analysis of age, race/ethnicity, and HIV status in LAC. It also offers detailed insights into how vaccination impacts different groups at risk for acquiring mpox, providing a more nuanced understanding of the spread and control of the disease.

## METHODS

We developed a microsimulation model with weekly cycles for MSM in LAC and simulated several counterfactual scenarios to explore variation in vaccination magnitude, timing, and distribution, as well as timing and magnitude of sexual risk reduction to mitigate transmission risk. We explored vaccine prioritization by race/ethnicity and by HIV status and examined the effect of initial reductions in sexual partnerships followed by rebounds. This approach allowed us to understand the potential outcomes if low sexual contact rates could not be sustained and to evaluate the robustness of vaccination policies.

### Model Structure and Dynamics

**Figure 1.**
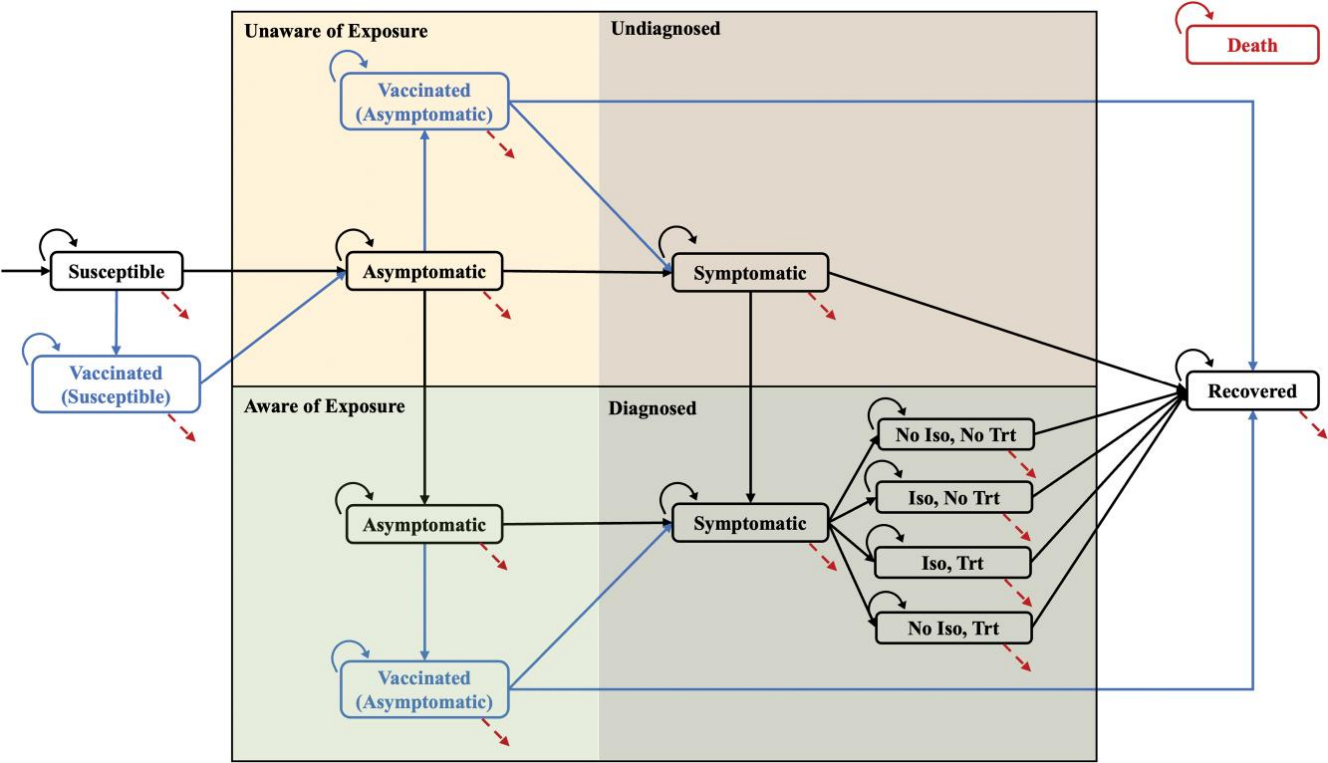
Model schematic. This figure illustrates the transitions between health and treatment states. Background colors mark awareness and diagnosis stages: yellow for “unaware of exposure” in the asymptomatic phase and “undiagnosed” in the symptomatic phase; green for “aware of exposure” and “diagnosed” counterparts, respectively. Arrows between boxes represent possible transitions, denoting a likelihood of moving from one state to another. Circular arrows represent a likelihood of remaining in the current state. Red dashed arrows from all health states represent a probability of mortality. All transition probabilities are dependent on individual age, race/ethnicity, and HIV status, to the extent data permitted. Abbreviations: Iso, isolation; Trt, treatment.

The simulation model captured mpox transmission, diagnoses, vaccination, isolation, and treatment dynamics differentiated by an individual's age, race/ethnicity, and HIV status. Individuals with different sociodemographic characteristics transition through these health states with probabilities dependent on their age, race/ethnicity, and HIV status (Figure 1). Once infected, individuals became asymptomatic and could progress to a symptomatic state, from which they could recover. Our model accounts for possible awareness of exposure among asymptomatic individuals. While unaware individuals progress to an undiagnosed state upon symptom development and may subsequently be diagnosed, those aware of their infection are assumed to be diagnosed promptly after symptoms appear. Vaccination can be administered before symptom onset as either prevention for susceptible individuals or as postexposure prophylaxis (PEP) for those exposed. Administering the vaccine within 4 days of possible exposure could preempt the onset of symptoms, allowing individuals to recover without becoming symptomatic [12]. Symptomatic individuals could receive treatment, which increased the likelihood of recovery, and/or be isolated to prevent transmission to susceptible individuals. All patients in our cohort had a chance to start treatment, as calculated using LAC treatment surveillance data [3]. Each week, new susceptible individuals entered the system at the start of the model cycle. Individuals could exit the system due to death by other causes at any state, and no deaths were attributed to mpox as there were only 2 mpox associated deaths in LAC as of November 2023 [3].

In line with evidence from the literature, the simulation assumes that transmission occurs exclusively during the symptomatic phase [6]. Current evidence also identifies sexual contact as the primary transmission pathway for mpox [6, 8–10], and our model captures infection solely through sexual interactions. To address variations in transmission rates among different subpopulations, we integrated a partnership mixing matrix. This matrix captured the likelihood of sexual partnership across diverse age and racial/ethnic groups, enabling our model to reflect some of the complexity of real-world sexual network interactions (Supplementary Data, Model Details and Inputs section).

Our model simulated HIV infections by including 36 new HIV cases weekly, distributed across various age and racial/ethnic groups, consistent with a prior published HIV model based in LAC [20]. The estimation of new HIV diagnoses was similarly derived, with further details in the Supplementary Data, Model Details and Inputs section. Crucially, our model recognizes that PWH have a higher probability of acquiring mpox compared with those who are HIV negative. This assumption is supported by data indicating that PWH comprised 45% of total mpox infections in the LAC region as of September 25, 2022 (as reported by LACDPH in our regular meetings). Additional evidence reinforcing this assumption is documented in other published works [5, 21].

Our simulation model began on July 3, 2022. It spanned 40 weeks, capturing disease dynamics until April 9, 2023.

### Initial Cohort

To establish the initial population for our simulation, we utilized data from a previously published paper that provide detailed insights on the size and demographics of the MSM population in LAC, including factors like age, race/ethnicity, and HIV status [20]. We then determined the mpox infection and vaccination statuses of this initial cohort, drawing on empirical data from the LACDPH (see the Initial Population section in Table 1). The initial cohort, categorized by age, race/ethnicity, and HIV status where available, was instrumental in accurately setting the baseline conditions for the first week (June 26–July 2, 2022) of our simulation.

**Table 1.** Selected Model Inputs

Parameter	Value	Source
Initial population		
LAC MSM count	262 912	[20]
Proportion of PWH	0.217	[20]
Proportion of HIV status aware PWH	0.869	[20]
No. of mpox asymptomatic	202	Calibrated
No. of mpox symptomatic	83	Assumption
No. of mpox symptomatic who are diagnosed	33	^d^
No. of vaccinated (1st dose)	176	^d^
Proportion of mpox infection who are PWH	0.45	^d^
Proportion of mpox infection by race^a^	[0.06, 0.34, 0.57]	^d^
Proportion of mpox infection by age^b^	[0.20, 0.60, 0.11, 0.09, 0.00]	^d^
Proportion of vaccination (1st dose) by race^a^	[0.057, 0.159, 0.784]	^d^
Proportion of vaccination (1st dose) by age^b^	[0.26, 0.50, 0.19, 0.05, 0.01]	^d^
Birth dynamics		
No. of new entrants as a proportion of simulated population in the prior year	0.0003	[22]
New entrants by race^a^	[0.099, 0.569, 0.332]	[23]
Transition probabilities: disease natural history		
P(aware | asymptomatic, HIV- and HIV status unaware PWH)	0.01	Assumption
P(aware | asymptomatic, HIV status aware PWH)	0.1	Assumption
P(diagnosed | symptomatic) by age^b^	[0.8, 0.9, 0.8, 0.9, 0.7]	Calibrated
P(asymptomatic -> symptomatic)	0.5	Calculated from [24]
P(asymptomatic -> recovered | vaccinated within a week)	0.43	Calculated from [12]
P(symptomatic -> recovered | no treatment)	0.28	Calculated from [24]
P(symptomatic -> recovered | treatment)	0.5	Calculated from [25]
Transition probabilities: vaccination, treatment, and isolation		
Vaccine efficacy (1st dose)	0.78	[26]
Vaccine efficacy (2nd dose)	0.85	[26]
P(getting 1st dose vaccination | susceptible or undiagnosed asymptomatic)		
Week 1	0.0002	^d^
Week 2	0.002	^d^
Week 3–8	0.025	^d^
Week 9 and after	0.005	^d^
P(getting 1st dose vaccination | aware, asymptomatic)	0.95	Assumption
P(getting 2nd dose vaccination | eligible people^c^)	0.5	Assumption
P(start treatment | diagnosed, symptomatic)	0.3	Assumption
P(being isolated | diagnosed, symptomatic)	0.2	Assumption
Calibration parameters		
Force of infection (week 1–5)	2.2	Calibrated
Force of infection (week 6 and after)^e^	0.7	Calibrated
Relative risk by race^a^	[1.5, 1, 0.8]	Calibrated
Relative risk (PWH vs HIV-)	[3.1, 1]	Calibrated
Relative risk by age (15–24, 25–44, 45–54, 55–100)	[0.37, 1.33, 1, 0.26]	Calibrated
Scaling factor for receiving 1st dose vaccination by race^a^ starting from week 3	[1, 0.65, 1.5]	Calibrated
Scaling factor for receiving 1st dose vaccination by age^b^ starting from week 3	[1, 2, 1.2, 1, 0.5]	Calibrated

Table 1 summarizes other key inputs used in our simulation. See the Supplementary Data, Model Details and Inputs section, for further details.

Abbreviations: LAC, Los Angeles County; LACDPH, Los Angeles County Department of Public Health; MSM, men who have sex with men; PWH, people with HIV.

^a^Race: Black, Hispanic, White.

^b^Age: 15–29, 30–39, 40–49, 50–59, 60–100.

^c^Eligible people: people who got first dose vaccination 4–6 weeks before and were susceptible or undiagnosed asymptomatic.

^d^Calculated from the data provided by LACDPH.

^e^This parameter is used to calibrate the probability of infection and indirectly captures the sexual partnership rate in the simulation.

### Model Inputs

The input parameters for the natural history and infection dynamics of the disease were collected from various sources, including published literature, empirical data, and expert knowledge.

In accordance with the vaccination protocol in LAC, individuals in the simulation were eligible for their second vaccine dose 4 weeks after receiving the initial dose [12]. We assumed this second dose could be received any time between 4 and 6 weeks after the first vaccination. It was expected that vaccine-induced protection for a dose would occur 2 weeks after receiving it [27].

We derived the vaccination rate for susceptible individuals from LAC data. Our model assumed that asymptomatic individuals who were unaware of their exposure had the same probability of receiving vaccination as the general susceptible population. Conversely, asymptomatic individuals who were aware of their infection were presumed to have a higher likelihood of being vaccinated, estimated at 95%. This assumption was based on the premise that people with a known exposure who are offered PEP have a high likelihood of accepting and receiving it. To reflect real-world trends, vaccination rates by race/ethnicity and age were calibrated starting from the third week of the simulation (described in the Calibration section below).

The likelihood of receiving an mpox diagnosis in our model was influenced by age, race/ethnicity, and HIV status (Table 1). We assumed that PWH who were aware of their HIV status were more proactive in seeking testing compared with all other MSM, thereby increasing their chances of mpox diagnosis.

We simulated mpox treatment using tecovirimat (TPOXX), as studies have shown that TPOXX is effective in reducing mpox symptoms [25]. Our model assumed that TPOXX treatment shortens the recovery time from 21 to 10 days.

To capture the dynamic changes in infection rates over time, as observed in empirical data, our model employed a calibration parameter known as the “force of infection” (FoI). This parameter adjusts the infection probability, allowing for fine-tuning of the transmission dynamics to match observed trends (see Table 1, Calibration Parameters, for parameter values). Group-specific calibration parameters further refined transmission rates, allowing our model outputs to closely match empirical data. More details on this process are available in the Calibration section.

### Calibration

We calibrated the model to match 19 targets over the first 12 weeks of the simulation. These targets were the number of diagnosed cases and vaccinated individuals broken down by age, race/ethnicity, and HIV status wherever possible. The calibration process involved adjusting age- and race-specific calibration parameters until our model outputs closely mirrored actual data trends in the 12-week calibration period. This detailed calibration approach enabled us to capture the nuanced variations in how the disease impacts different population segments. More information on the calibration process, including results, parameter values, and methods used, can be found in the Supplementary Data, Calibration and Validation section.

### Validation

We validated the model by comparing model outputs on the number of diagnoses, vaccinations, and people on treatment against available empirical data that were not used in calibration. The model's predictions for the number of diagnoses and vaccination, categorized by age, race/ethnicity, and HIV status, continued to align closely with observed empirical trends even after the 12-week calibration period. Noncalibrated outcomes such as treatment and diagnosed cases among PWH by race/ethnicity and age also matched the empirical data. This concordance demonstrated the model's robustness and its ability to accurately predict disease dynamics over an extended period. See more details in the Supplementary Data, Calibration and Validation section.

### Model Scenarios

We used multiple counterfactual scenarios to analyze the impact of various factors on mpox spread, including vaccination magnitude, timing, prioritization, timing of the reduction in sexual partnerships, and the potential for a rebound in sexual partnerships (see Supplementary Table 4 for a list of scenarios). We then compared these scenario outcomes with those of the status quo.

We evaluated how different levels of vaccine uptake affected disease dynamics through 3 scenarios: “no vaccination” (no vaccination), “50% vaccination rate” (half the status quo first-dose vaccination rate), and “200% vaccination rate” (double the status quo first-dose vaccination rate). To understand the impact of vaccination timing, we created scenarios where vaccine administration either occurred 2 weeks earlier or was delayed by 2, 4, or 8 weeks compared with the status quo. This approach enabled us to understand the influence of both the magnitude and timing of vaccinations on the progression of the outbreak.

Building upon our status quo analysis, which followed empirically observed vaccine uptake patterns, we explored the implications of prioritizing vaccinations for different risk groups, such as PWH, who are more susceptible to mpox, and specific racial/ethnic groups (Black, Hispanic, or White). In these scenarios, vaccines were initially allocated exclusively to these prioritized groups, following the same weekly dosage as in the status quo. Once all individuals in these groups were vaccinated, the remaining doses were distributed equitably among other demographic groups.

We also examined scenarios where the reduction in transmission occurred either 3 weeks earlier or 3 weeks later than observed in the status quo (August 2022). Given the uncertainties about sustained sexual risk reduction, we included scenarios accounting for rebounds in the transmission rate to help us assess the necessary duration and extent of reductions in sexual partnerships needed to prevent a potential future outbreak. We investigated scenarios where a rebound in sexual partnerships occurred in October, November, or December, with the intensity set at either half or at the same level as observed before August 2022. This approach allowed us to understand the timing and impact of sexual risk reduction on transmission dynamics more clearly. The extent of rebound levels was modeled in the simulation by adjusting the FoI, which scales the probability of infection. Importantly, this adjustment does not imply a direct 1:1 relationship with transmission rates (see Supplementary Data, Model Scenarios section, for more details). Additionally, we applied different vaccination rates to the scenario where transmission levels returned to the pre­–August 2022 levels in October to examine the effectiveness of vaccination strategies under a scenario of heightened risk.

## RESULTS

### Effect of Vaccine Magnitude

Under the status quo, we observed that a total of 130 490 vaccine doses (with a 95% uncertainty interval [UI] of 129 486–131 468) were administered over 40 weeks. A reduction to half the status quo vaccination rate led to administering 58 784 fewer doses, resulting in an increase of 194 (UI, 96–294) cumulative cases, representing an 8% increase. On the other hand, doubling the vaccination rate required an additional 88 995 doses, which was associated with a reduction of 296 (UI, 200–375) cases, representing a 13% decrease. In an extreme hypothetical scenario where no vaccines were distributed, our model projected an increase of 656 (UI, 548–777) cumulative cases, which represents a 28% surge.

**Figure 2.**
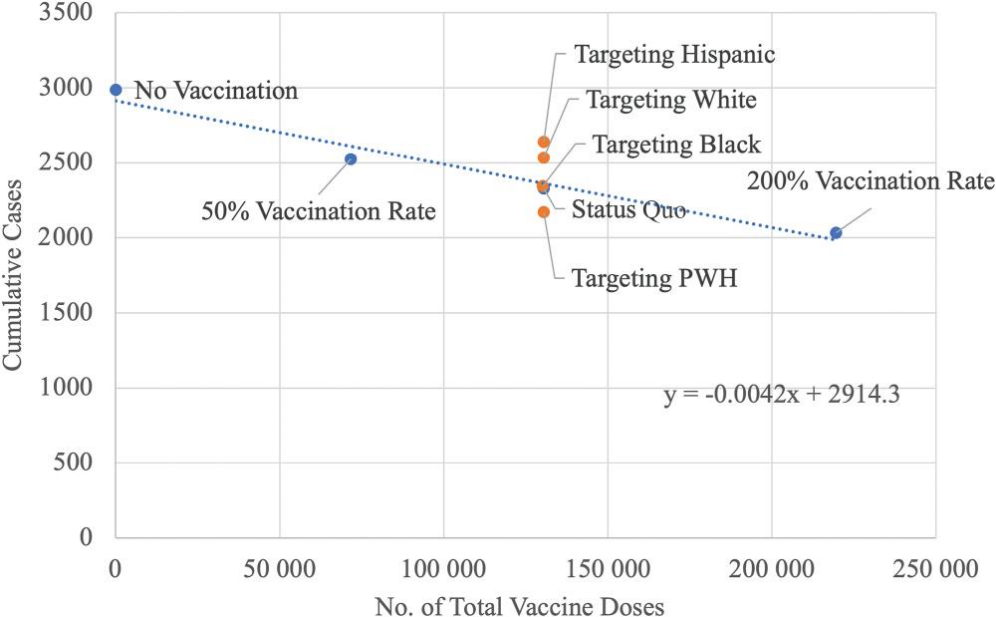
Cumulative cases over 40 weeks. As expected, cumulative cases generally decline with additional vaccination doses, although we find that some strategies (eg, targeting vaccination by race/ethnicity) may result in a higher number of cumulative cases than the status quo.

The simulation outputs reveal an inverse relationship between the number of vaccine doses and cumulative cases over the 40-week period, suggesting that higher vaccine distribution is linked to a decrease in cases (Figure 2), as expected. However, for every 10 000 vaccine doses, only ∼42 additional cases are averted (further details in the Supplementary Results).

### Effect of Vaccination Timing

**Figure 3.**
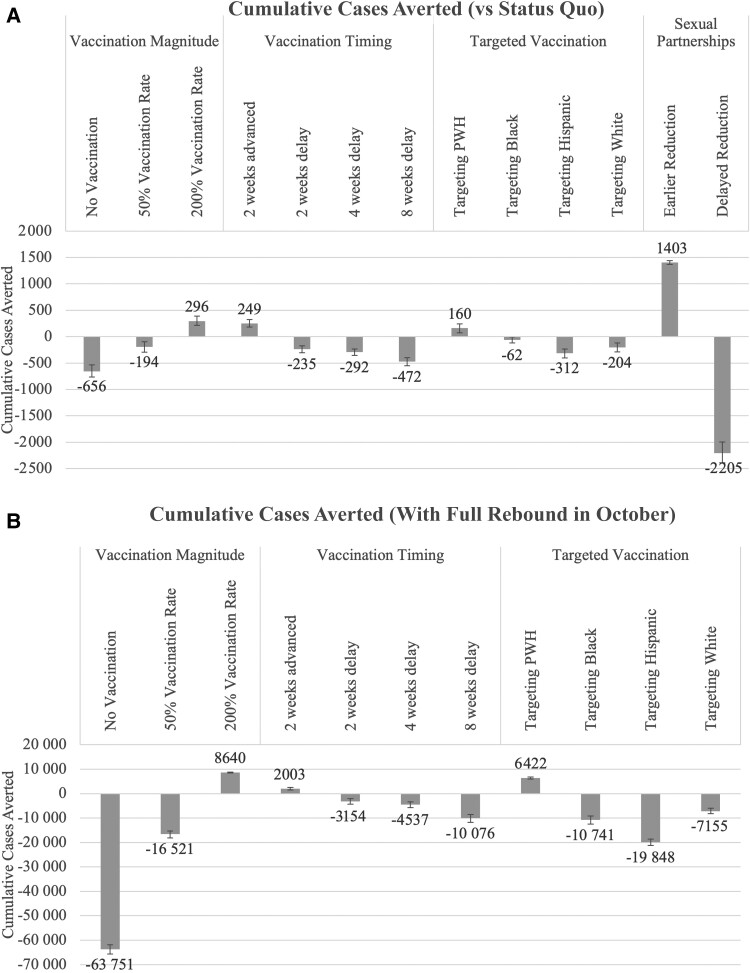
Cumulative cases averted under different scenarios.

Our simulation revealed that initiating the vaccination campaign 2 weeks earlier could have led to 249 (UI, 170–315) fewer cumulative mpox cases, an 11% decrease from the status quo. This suggests that an earlier distribution of vaccines could have reduced the number of new cases. Conversely, starting 2 weeks late led to an additional 235 (UI, 166–298) cases, a 4-week delay resulted in 292 (UI, 231–350) more cases, and an 8-week delay caused an increase of 472 (UI, 391–546) cases, or a 10%, 13%, and 20% increase in cases, respectively (Figure 3A). These findings underscore the time-sensitive nature of vaccine administration. Distributing vaccines 2 weeks earlier resulted in a similar number of cases averted compared with doubling the vaccination rate (2% difference in cumulative cases) but required 86 109 fewer doses.

### Targeted Vaccination Efforts

In scenarios exclusively administering vaccines to either PWH or specific racial/ethnic groups (Black, Hispanic, White), we found that targeting PWH resulted in a 7% (160 cases; UI, 79–247) decrease in cumulative cases compared with the status quo. When vaccines were allocated based on race/ethnicity, we observed a statistically significant increase in the number of cumulative cases when vaccines were only allocated to Hispanic or White MSM, and a slight increase when allocated to only Black MSM (Figure 3A).

### Timing of Reduction in Sexual Partnerships

Modeling the impact of timing in sexual risk reduction revealed significant effects on mpox spread. Delaying the reduction of sexual partnerships by 3 weeks resulted in a significant surge in cases, with 2205 (UI, 2020–2417) additional cases observed, representing a 95% increase (almost twice as many cases). In contrast, 3 weeks earlier in behavioral changes led to 1403 (UI, 1368–1436) fewer cases, a 60% decrease from the status quo (Figure 3A).

### The Effect of a Rebound in Prior Sexual Partnership Levels

**Figure 4.**
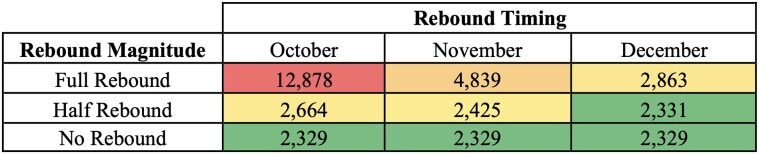
Cumulative Cases With Rebound and No Rebound.

In scenarios modeling a rebound in sexual partnerships to pre–August 2022 level at half or full intensity during October, November, or December of 2022, we observed varied impacts on mpox spread (Figure 4). If the rebound in sexual partnerships was half of the pre-August level and occurred in November or December, the increase in cumulative cases was relatively small: an increase of 96 (UI, 1–198) cumulative cases for November and 2 (UI, −88 to 103) for December. A half rebound occurring in October led to a more substantial increase of 334 (UI, 234–441) cases. However, the effects of a full rebound were considerably more pronounced across all 3 timing scenarios: 533 (UI, 362–700) additional cases for December, 2509 (UI, 2130–2913) for November, and, markedly,10 548 (UI, 9639–11 582) additional cases in October.

While the half rebound in October resulted in a significant increase in cumulative cases, the incidence trends over time indicate that all half rebound scenarios, including that of October, eventually led to a gradual return to the status quo. In contrast, incident cases in full rebound scenarios diverged over time markedly. See the incidence over time trend for rebound scenarios in Supplementary Figure 3.

### Vaccination Scenarios Under Full Transmission Rebound in October

We also wanted to understand the incremental effect of vaccination policies if there was a full rebound in transmission in October. Given the rebound in transmission rates, we compared outcomes with the vaccination scenarios described above with those with vaccination rates observed in the status quo. Halving the vaccination rate resulted in 16 521 (UI, 15 311–18 094) additional cases, representing a 128% increase in cumulative cases. Conversely, doubling the vaccination rate led to 8640 (UI, 8462–8820) fewer cases, representing a 67% decrease, and with no vaccination, there were 63 751 (UI, 61 837–65 625) additional cases, representing a 496% increase. When targeting PWH, there were 6422 (UI, 6044–6836) fewer cases, representing a 50% reduction. However, targeting any racial/ethnic group resulted in significantly more cumulative cases (Figure 3B). These outcomes were consistent with our findings in the nonrebound scenario but with larger effect sizes, as expected due to the larger underlying transmission rate.

## DISCUSSION

Our study reaffirms the importance of timely and extensive vaccination coverage in controlling mpox transmission. However, the modest increase in averted cases with additional vaccine doses suggests that reliance on vaccination alone may not lead to substantial reductions in cases. By contrast, a 3-week delay in sexual risk reduction, such as reducing sexual partnerships, could potentially result in a 95% increase in cumulative cases. This highlights that timely behavioral interventions could be even more crucial than vaccination in reducing the overall burden of mpox, a conclusion that aligns with prior research [28–30].

Our simulation results also underscore the critical role of swift and proactive vaccination distribution in controlling the spread of mpox. Delays or insufficiencies in vaccination efforts were found to significantly escalate the number of mpox cases. An 8-week postponement in vaccination distribution resulted in a 20% increase in cases over 40 weeks, while no vaccination resulted in a 28% increase and halving the vaccination rate (45% fewer doses distributed) resulted in an 8% increase in cases. These findings emphasize the need for prompt and adequate vaccination strategies to mitigate the overall impact of disease outbreaks. It is therefore particularly important to prevent or mitigate delays in vaccine administration, which can occur due to logistical or administrative challenges.

The pivotal role of sexual risk reduction in controlling mpox is further underscored by our simulations of scenarios where transmission rates rebounded in the fall after they dropped in August 2022. These results highlight the critical influence of both the timing and extent of resumed sexual behaviors on mpox transmission. We observed that a rebound in transmission rates later in the year had a smaller impact, and a partial return to pre-August transmission levels in November or December did not significantly affect the overall cumulative case counts. However, large rebounds in transmission rates could lead to an outbreak, particularly if they occur earlier—the scenario with a full rebound in October resulted in a 453% increase in cases compared with the status quo over the 40-week time horizon. These findings emphasize the need for continued guidance and culturally tailored public health messaging for MSM with information about sexual risk reduction [31].

Furthermore, our study indicates that targeting specific population subgroups must be done with care. Targeting MSM by race/ethnicity may increase cumulative cases, with larger effects under transmission rebound scenarios. However, prioritizing vaccinations for PWH could effectively reduce mpox cases. This aligns with findings from other studies conducted in different regions or among smaller groups [28–30]. It also underscores the importance of leveraging existing HIV service providers to vaccinate MSM with HIV about mpox.

Our study, while providing valuable insights into the control of mpox in LAC, has several limitations. Input values were gathered from disparate sources, which could result in inconsistencies in our inputs. However, all inputs were vetted by experts at the LACDPH. Many parameters are uncertain, but we calibrated our model to empirical data across several measures to increase model confidence. We assumed no reinfection in our model, as antibodies produced after initial infection help protect the person from getting reinfected [9]. However, other studies suggest that natural immunity is not fully protective against mpox, although it may convey reduced disease duration and severity [32]. Additionally, we assume that asymptomatic individuals do not transmit the disease, although some studies indicate potential transmission during the asymptomatic phase [33]. It is important to note that these results are specific to mpox Clade IIb and may not apply to Clade I, which, while not currently reported in the United States, could potentially arrive in the future [34, 35]. The model only considered transmission through sexual contacts as this was the primary route of transmission; however, the disease can be transmitted through direct contact with lesions [36]. We also assumed transmission rates were homogeneous within age/race groups. In reality, partnership rates are determined by individual behavioral characteristics, and studies show that cases have been predominantly in MSM who have greater numbers of sexual partners [14, 37]. In the model, all MSM had the chance to start treatment and reduce the number of days to recovery; however, treatment may not be available to or appropriate for everyone, and the effectiveness of treatment is unknown [38]. Our study also does not consider the waning efficacy of vaccines or natural immunity over time, which could have significant implications for long-term disease control [39]. In addition, the benefits of intervention measures should always be evaluated in conjunction with their potential costs, such as the need for additional vaccine doses, which we do not consider in this study. A balance between effective intervention strategies and resource allocation is paramount in controlling the mpox outbreak effectively, and this could be a fruitful direction of future study.

Despite these limitations, we believe our general findings could be applied to future outbreaks of mpox or other infectious diseases, particularly among MSM within Los Angeles County. Policy-makers could use this model as a foundation for developing targeted interventions in different locations. Further research is warranted to identify the optimal combination of policies, including vaccination strategies and behavioral interventions, to effectively control the spread of mpox and similar diseases.

## Supplementary Data

Supplementary materials are available at *Open Forum Infectious Diseases* online. Consisting of data provided by the authors to benefit the reader, the posted materials are not copyedited and are the sole responsibility of the authors, so questions or comments should be addressed to the corresponding author.

## Supplementary Material

ofae401_Supplementary_Data
