# Supplementary material for: A Microsimulation Model of Mpox in Los Angeles County: Implications for Future Disease Prevention and Control Strategies among Men Who Have Sex with Men
Source: Open Forum Infect Dis. 2024 Jul 17;11(Suppl 2):S137–45. doi: 10.1093/ofid/ofae401 (PMC11477083; doi:10.1093/ofid/ofae401)
Supplement: ofae401_Supplementary_Data [file ofae401_supplementary_data.docx]

**Appendix for**

**A Microsimulation Model of Monkeypox (Mpox) in Los Angeles County (LAC): Implications for Future Disease Prevention and Control Strategies among Men Who Have Sex with Men (MSM)**

By Citina Liang, Sze-chuan Suen, Chenglin Hong, Andrea Kim, Rita Singhal, Paul Simon, Mario Perez, Ian W. Holloway

Table of Contents

[**Model Details and Inputs** 2](#_Toc164771559)

[**Initial Cohort Generation** 2](#_Toc164771560)

[**Model Description and Transition Probabilities** 4](#_Toc164771561)

[**Transmission Rate** 6](#_Toc164771562)

[**Calibration and Validation** 7](#_Toc164771563)

[**Calibration** 7](#_Toc164771564)

[**Validation** 8](#_Toc164771565)

[**Model Scenarios** 13](#_Toc164771566)

[**Results** 14](#_Toc164771567)

[**Vaccination Scenarios** 14](#_Toc164771568)

[**Sexual Behavior Rebound Scenarios** 16](#_Toc164771569)

[**Full Transmission Rebound in October Scenario with Vaccination strategies** 18](#_Toc164771570)

# **Model Details and Inputs**

## ***Initial Cohort Generation***

Our simulation's initial population utilized a representative demographic profile by age, race/ethnicity, and HIV status for the men who have sex with men (MSM) cohort of Los Angeles County (LAC) in the year 2022. This demographic data was simulated based on an individual-level HIV model detailed in a previous study.^1^

The simulation began on June 26, 2022, coinciding with the report of the first LAC mpox case around mid-May. Consequently, our initial cohort included individuals who were either already infected with mpox or vaccinated. The infected segment of the cohort was further categorized into asymptomatic or symptomatic groups. We assumed that no one in the initial cohort was diagnosed if they were asymptomatic, and that none had recovered yet. The number of asymptomatic individuals was estimated through calibration, with the methodology and outcomes detailed in the Appendix Calibration and Validation section.

Drawing on empirical data from the Los Angeles County Department of Public Health (LACDPH), we established age, race/ethnicity, and HIV status-specific mpox infection and vaccination statuses for our initial cohort. Additionally, we determined age and race/ethnicity-specific HIV infection rates from a prior HIV study.^1^ This detailed categorization by race/ethnicity, age, and HIV status was crucial in establishing accurate baseline conditions for the first week of the simulation.

See details for parameters used in the initial cohort in Appendix Table 1.

| **Appendix Table 1: Model Inputs** | | |
| --- | --- | --- |
| **Parameter** | **Value** | **Source** |
| Initial Population | | |
| LAC MSM count | 262,912 | ^1^ |
| Proportion of PWH | 0.217 | ^1^ |
| Proportion of HIV-status aware PWH | 0.869 | ^1^ |
| No. of mpox asymptomatic | 202 | Calibrated |
| No. of mpox symptomatic | 83 | Assumption |
| No. of mpox asymptomatic that are diagnosed | 0 | Assumption |
| No. of mpox symptomatic that are diagnosed | 33 | **** |
| No. of vaccinated (1^st^ dose) | 176 | **** |
| Proportion of mpox infection that are PWH | 0.45 | **** |
| Proportion of mpox infection by race* | [0.06, 0.34, 0.57] | **** |
| Proportion of mpox infection by age** | [0.20, 0.60, 0.11, 0.09, 0.00] | **** |
| Proportion of vaccination (1^st^ dose) by race* | [0.057, 0.159, 0.784] | **** |
| Proportion of vaccination (1^st^ dose) by age** | [0.26, 0.50, 0.19, 0.05, 0.01] | **** |
| Birth Dynamics | | |
| No. of new entrants as a proportion of simulated population in the prior year | 0.0003 | ^2^ |
| New entrants by race* | [0.099, 0.569, 0.332] | ^3^ |
| Transition Probabilities: Disease Natural History | | |
| P(diagnosed \| asymptomatic, HIV-negative and HIV-status unaware PWH) | 0.01 | Assumption |
| P(diagnosed \| asymptomatic, HIV-status aware PWH) | 0.1 | Assumption |
| P(diagnosed \| symptomatic) by age** | [0.8, 0.9, 0.8, 0.9, 0.7] | Calibrated |
| P(asymptomatic -> symptomatic) | 0.5 | Calculated from ^4^ |
| P(asymptomatic -> recover \| vaccinated within a week) | 0.43 | Calculated from ^5^ |
| P(symptomatic -> recovered \| no treatment) | 0.28 | Calculated from ^4^ |
| P(symptomatic -> recovered \| treatment) | 0.5 | Calculated from ^6^ |
| Transition Probabilities: Vaccination, Treatment, and Isolation | | |
| Vaccine efficacy (1^st^ dose) | 0.78 | ^7^ |
| Vaccine efficacy (2^nd^ dose) | 0.85 | ^7^ |
| Average No. of days for vaccine immune response (1^st^ dose) | 14 | ^5^ |
| Average No. of days for vaccine immune response (2^nd^ dose) | 14 | ^5^ |
| P(getting 1^st^ dose vaccination \| susceptible or asymptomatic unaware of exposure) | | |
| Week 1 | 0.0002 | **** |
| Week 2 | 0.002 | **** |
| Week 3-8 | 0.025 | **** |
| Week 9 and after | 0.005 | **** |
| P(getting 1^st^ dose vaccination \| aware of exposure, asymptomatic) | 0.95 | Assumption |
| P(getting 2^nd^ dose vaccination \| eligible people***) | 0.5 | Assumption |
| P(start treatment \| diagnosed, symptomatic) | 0.3 | Assumption |
| P(being isolated \| diagnosed, symptomatic) | 0.2 | Assumption |
| Other Transition Probabilities | | |
| Partnership Mixing Matrix | See details from source | ^1^ |
| Weekly No. of Partners by age (15-19, 20-24, 25-34, 35-44, 45-54, 55-75, 76-100) | [0.154, 0.231, 0.231, 0.154, 0.231, 0.154, 0] | ^1^ |
| Probability of natural death by age | See details from source | ^1^ |
| Calibration Parameters | | |
| Force of Infection (week 1-5) | 2.2 | Calibrated |
| Force of Infection (week 6 and after) | 0.7 | Calibrated |
| Relative risk by race* | [1.5, 1, 0.8] | Calibrated |
| Relative risk (PWH vs. HIV-) | [3.1, 1] | Calibrated |
| Relative risk by age (15-24, 25-44, 45-54, 55-100) | [0.37, 1.33, 1, 0.26] | Calibrated |
| Scaling factor for receiving 1^st^ dose vaccination by race* starting from week 3 | [1, 0.65, 1.5] | Calibrated |
| Scaling factor for recieving1^st^ dose vaccination by age* starting from week 3 | [1, 2, 1.2, 1, 0.5] | Calibrated |
| *Race: Black, Hispanic, White | | |
| **Age: 15-29, 30-39, 40-49, 50-59, 60-100 | | |
| ***Eligible people: people who got 1^st^ dose vaccination, 4-6 weeks after 1^st^ vaccination, and susceptible or undiagnosed asymptomatic | | |
| ****Calculated from the data provided by LACDPH | | |

## ***Model Description and Transition Probabilities***

Our model depicted a weekly progression of mpox infection and vaccination, beginning with individuals aged 15 entering the system. The model delineated several states—susceptible, asymptomatic, symptomatic, and recovered—as depicted in Manuscript Figure 1. Individuals were infectious during Symptomatic state primarily through sexual contacts.^8,8–12^ The model allows for vaccination before the onset of symptoms; notably, symptomatic individuals who were diagnosed may undergo isolation or treatment, thereby mitigating the transmission risk or altering recovery trajectories. Each week, unvaccinated susceptible individuals may receive their initial vaccine dose, with probabilities adjusted for age and race/ethnicity. Asymptomatic individuals, irrespective of diagnostic status, were also eligible for vaccination within the simulation. We assumed that diagnosed individuals were highly likely to pursue vaccination (refer to Appendix Table 1 for probabilities). A key advantage of vaccination during the asymptomatic state was the potential for individuals to recover without progressing to the symptomatic state, thus precluding transmission.^5^

Post-vaccination, individuals achieved full immunity within two weeks, with the first dose's efficacy at 78% and the second dose's at 85%. Therefore, even vaccinated individuals retained a residual risk of infection due to the vaccine's non-absolute efficacy.

Under the constant transition rate assumption, the model posited that the time between health states is exponentially distributed. Hence, the transition rate ($\alpha)$reflects the inverse of the mean duration (in weeks) between health states. The likelihood of transitioning within the ensuing week was expressed as $1-e^{-\alpha}$. For example, consider calculating the probability of transitioning from asymptomatic to symptomatic. The incubation period for the condition ranges from 3 to 17 days.^4^ Taking the average of this range gives us an average incubation period of 10 days, equivalent to $\frac{10}{7}$​ weeks. Given this, the transition rate ($\alpha$) is determined as the inverse of the mean duration in weeks, or $\frac{7}{10}$​. Therefore, using our formula, the likelihood of transitioning from asymptomatic to symptomatic within a week is calculated as $1-e^{-\frac{7}{10}}$, which approximately equals 0.5. Probabilities for transitions from asymptomatic to symptomatic, asymptomatic to recovery upon vaccination within a week, and symptomatic to recovery without treatment were also computed using the same approach (see Appendix Table 1 for these probabilities).

The simulation incorporated HIV infection and diagnosis dynamics, informed by a previously published model of HIV among MSM in LAC, to ascertain the incidence and diagnostic characteristics for HIV in 2022.^1^ Given that the referenced model operated on an annual basis, we interpolated these data to a uniform weekly distribution for the simulation period. Moreover, we had stratified HIV-positive individuals by age and race/ethnicity to maintain demographic precision (see Appendix Tables 2a and 2b for a detailed demographic stratification).

| **Appendix Table 2a. No. of People of Weekly HIV Infection by Demographic Groups** | | | | |
| --- | --- | --- | --- | --- |
|  | Age | | | |
| Race | 15-29 | 30-49 | 50-64 | 65+ |
| Black | 3 | 3 | 2 | 1 |
| Hispanic | 6 | 7 | 5 | 2 |
| White | 2 | 2 | 2 | 1 |

| **Appendix Table 2b. No. of People of Weekly HIV diagnosis by Demographic Groups** | | | | |
| --- | --- | --- | --- | --- |
|  | Age | | | |
| Race | 15-29 | 30-49 | 50-64 | 65+ |
| Black | 2 | 3 | 2 | 0 |
| Hispanic | 5 | 7 | 4 | 1 |
| White | 2 | 3 | 2 | 0 |

## ***Transmission Rate***

In our model, the probability that an individual would be infected, denoted as $P(Infection)$, was derived from the interaction of several factors that reflected the demographic diversity and behavioral patterns of the population. The equation below captures this interaction:

$$P\left( Infection \right)=1-\prod_{d_{p}\in D} \left( 1-\alpha\gamma_{a}\beta_{d}\frac{I_{d_{p}}}{N_{d_{p}}} \right)^{P_{d}M_{d_{p}}}$$

To scale the dynamics of disease transmission within demographic groups, we introduced the calibration parameter Force of Infection (FoI), $\alpha,$ into the equation. The FoI was designed to modify the impact of infected individuals on the susceptible population within their respective demographic group.

Where:

$D:$ set of possible demographic groups

$d_{p}:$ demographic of the susceptible population

$I:$ infected group that can transit in demographic group of partners (exclude those who are isolated)

$\alpha:$ Force of Infection (FoI)

$\beta_{d}$: calibration parameter for race/ethnicity group d, where d = Black, Hispanic, White

$\gamma_{a}$: calibration parameter for age groups a, where a = 15-24, 25-34, 35-44, 45-100

$N_{d_{p}}:$ number of people in partner demographic group $d_{p}$

$P_{d}:$ number of partners an individual in the demographic group d have

$M_{d_{p}}:$ probability of susceptible individual mixing with partner demographic group $d_{p}$

For each susceptible demographic group $d_{p}$ within the set of all possible groups $D$, we calculated the probability of not becoming infected. This was done by taking the product over all $d_{p}$ groups of the term $\left( 1-\alpha\gamma_{a}\beta_{d}\frac{I_{d_{p}}}{N_{d_{p}}} \right)^{P_{d}M_{d_{p}}}$. In this equation, $\alpha$ represented the FoI, establishing the baseline risk of disease spread. $\beta_{d}$ is a calibration parameter specific to each race/ethnicity group $d$, where $d$ can be Black, Hispanic, or White, which allows the model to reflect observed disparities in infection rates among different race/ethnicity groups. $\gamma_{a}$ adjusts the risk based on age group $a$, accounting for the varying susceptibility and social dynamics of age brackets 15-24, 25-34, 35-44, and 45-100. The term $I_{d_{p}}$represents the number of infected individuals within the partner demographic group $d_{p}$ who can transmit disease, excluding those who are isolated. $N_{d_{p}}$ is the total number of individuals in the partner demographic group. $p_{d}$ specifies the average number of partners that an individual in demographic group $d$ is likely to have, and $M_{d_{p}}$ represents the mixing probability with the partner demographic group $d_{p}$.

By computing this product, we assessed the cumulative likelihood that a person would not encounter an infection across all their interactions with others. As FoI and the number of infected individuals in the population increase, the product decreases, and consequently, $P(Infection)$ increases, indicating a higher overall risk of infection.

# **Calibration and Validation**

## ***Calibration***

To determine the initial asymptomatic population and the FoI for our mpox model, we applied a grid search method for the calibration parameters for the first five weeks and separately for week five onwards. Initially, we explored various starting values for number of asymptomatic individuals in the initial cohort—128, 145, 156, 165, 185, 193—and FoI values for the first five weeks—2.1, 2.2, 2.3, 2.4, 2.5—resulting in 35 unique combinations. For each scenario, we calculated the sum of squared errors (SSE) using the metric number of diagnosed infections and identified the six scenarios with the lowest SSE (see Appendix Table 3a).

| **Appendix Table 3a. Sum of Square Errors for Calibration Scenarios** | | | | | | | |
| --- | --- | --- | --- | --- | --- | --- | --- |
|  | **Number of asymptomatic in the initial cohort** | | | | | | |
| **Force infection  ≤ Week 5** | 128 | 145 | 156 | 165 | 185 | 193 | 202 |
| 2.1 |  |  |  |  |  |  | 2812 |
| 2.2 |  |  | 6860 | 4107 | 4259 | 2683 | 606 |
| 2.3 |  | 6763 | 5878 | 4219 | 874 | 1120 | 696 |
| 2.4 | 9224 | 4054 | 3455 | 2583 | 367 | 1003 |  |
| 2.5 | 4590 | 1715 | 1814 | 1422 |  |  |  |

| **Appendix Table 3b. Sum of Square Errors for Calibration Scenarios** | | | | | | |
| --- | --- | --- | --- | --- | --- | --- |
|  | **Number of asymptomatic in the initial cohort** | | | | | |
| **Force infection > Week 5** | (2.3, 185) | (2.4, 185) | (2.3, 193) | (2.4, 193) | (2.2, 202) | (2.3, 202) |
| 0.6 | 11934 | 6898 | 7566 | 6144 | 7948 | 7096 |
| 0.7 | 5831 | 5580 | 4471 | 5187 | 3258 | 4284 |
| 0.8 | 5012 | 3672 | 5363 | 6466 | 4114 | 6151 |
| 0.9 | 8380 | 5520 | 6304 | 10876 | 6342 | 9641 |

Subsequently, we conducted a second grid search for the FoI from week five onwards, considering values of 0.6, 0.7, 0.8, 0.9. This created 24 additional scenarios. After running these simulations and assessing the SSE from the simulations, we selected the optimal parameters: 202 initial asymptomatic individuals, an FoI of 2.2 for weeks one to five, and an FoI of 0.75 for weeks six and beyond for the status quo scenario (see Appendix Table 3b).

In calibrating diagnosis and vaccination statuses, we refined our parameters at both a general level and within specific demographics, categorized by race/ethnicity and age. We defined five age brackets—15-29, 30-39, 40-49, 50-59, 60-100—each aligned with county mpox data. We calibrated these parameters to fit empirical data for the first 12 weeks of the 40-week simulation. This process involved changing the relative risk of mpox infection for racial groups, with Blacks experiencing higher risk and Whites lower risk than the average, we changed the calibration parameters accordingly. A similar process was followed to identify age group-specific risks. The outcomes for calibration parameters of this calibration process are outlined in Appendix Table 1 Calibration Parameters.

We considered a comprehensive suite of 19 calibration targets, including the number of mpox diagnoses, mpox diagnosis status by race (Black, Hispanic, and White), mpox diagnosis status by age (15-29, 30-39, 40-49, 50-59, 60-100), mpox diagnosis status by HIV status, number of vaccinations, vaccinations by race (Black, Hispanic, and White), and vaccinations by age (15-29, 30-39, 40-49, 50-59, 60-100). These targets were meticulously selected based on data provided by the county. The calibration results are depicted in Figures 1 a-e, g-j.

## ***Validation***

We performed internal validation of our model by comparing its predictions with empirical data that was not utilized during the calibration process. This comparison encompassed 23 targets, which included: the number of diagnosis of mpox, the number of diagnosis status of mpox by race (Black, Hispanic, and White), the number of diagnosis status of mpox by age (15-29, 30-39, 40-49, 50-59, 60-100), the number of diagnosis status of mpox by HIV status, number of vaccinated, number of vaccinated by race (Black, Hispanic, and White), the number of vaccinated by age (15-29, 30-39, 40-49, 50-59, 60-100), the number of treated cases, the number of diagnosed cases that are PWH by race (Black, Hispanic, and White), the number of diagnosed cases that are PWH by age (15-29, 30-39, 40-49, 50-59, 60-100).

The validation process entailed a visual comparison of the model's predictions with empirical trends through graphical representations. As illustrated in Appendix Figure 1 a, c, d, f-j, our model's predictions closely aligned with the empirical data well beyond the calibration period, demonstrating the model’s robustness and accuracy.

**Appendix Figure 1 a-j.**

Note: solid line represents calibration result and dashed line represents validation result.

# **Model Scenarios**

Appendix Table 4 provides an overview of model scenarios explored in our study. The scenarios are grouped based on several factors: the extent of vaccination coverage, the timing of vaccination rollouts, the specificity of vaccination targeting by demographic groups, and the timing of reductions in sexual partnership rates.

To model the different extent of sexual partnership rebound in our scenarios, we changed the probability of infection by adjusting the FoI. Specifically, for a full rebound, we set the FoI to 2.2, same as the value prior to the reduction in sexual partnerships in August 2022. For a half rebound, we set it to 1.1, which is half of the pre-reduction value. We maintained the FoI at 0.7, the post-reduction value, for scenarios with no rebound.

Manuscript Table 2 displays the projected cumulative incidence of disease over a 40-week period under different magnitude of rebound (either full or half) occurring in October, November, and December. This simulated results particularly emphasizes the significant impact of a full rebound in sexual partnership rates during October. To further understand the implications of these rebounds, we overlaid vaccination strategies onto this rebound scenario, assessing the robustness and providing insights into the effectiveness of these strategies in mitigating outbreak severity.

| Appendix Table 4: Model Scenarios | |
| --- | --- |
| Vaccination Magnitude | |
| No vaccination | No Vaccination |
| 50% vaccination rate | 50% of the probability of getting 1st dose vaccine compared to status quo |
| 200% vaccination rate | 200% of the probability of getting 1st dose vaccine compared to status quo |
| Vaccination Timing | |
| 2 weeks advanced | 2 weeks earlier of the vaccination distribution compared to the status quo |
| 2 weeks delay | 2 weeks later of the vaccination distribution compared to the status quo |
| 4 weeks delay | 4 weeks later of the vaccination distribution compared to the status quo |
| 8 weeks delay | 8 weeks later of the vaccination distribution compared to the status quo |
| Targeted Vaccination | |
| Targeting PWH | Same number of doses as status quo are distributed and prioritized to PWH |
| Targeting Black | Same number of doses as status quo are distributed and prioritized to Black |
| Targeting Hispanic | Same number of doses as status quo are distributed and prioritized to Hispanic |
| Targeting White | Same number of doses as status quo are distributed and prioritized to White |
| Sexual Partnerships Reduction Timing | |
| Earlier reduction | 3 weeks earlier of the sexual reduction compared to status quo |
| Delayed reduction | 3 weeks delay in the sexual reduction compared to status quo |

# **Results**

## ***Vaccination Scenarios***

Appendix Figure 2a shows the incident cases over time for various vaccination scenarios; Appendix Figure 2b shows a similar plot for targeted vaccination scenarios.

**Appendix Figure 2**

## ***Sexual Behavior Rebound Scenarios***

Manuscript Table 2 illustrates the effects of a “Full Rebound” in sexual partnerships, where we see a marked increase in cases, particularly in October. The “Half Rebound” scenario results in a moderate increase in cases, showing a slight decrease as the year progresses. In contrast, the “No Rebound” scenario maintains a consistent incidence across all three months, indicating stability when the rates of sexual partnerships do not increase. This table effectively captures the variance in disease spread due to changes in social behavior patterns post-intervention.

Appendix Figure 3 illustrates the impact of various rebound scenarios on the number of new mpox cases over time. In scenarios where sexual partnership rates rebound by half, we observe a modest increase in incident cases. Even when a full rebound occurs in December, the surge in cases is not particularly dramatic. However, when there is a full rebound take place in October or November, the figure clearly shows a significant escalation in the disease's spread. These observations are in line with the data presented in Manuscript Table 2, which details the cumulative incidence over a 40-week period under these differing rebound scenarios.

**Appendix Figure 3**

s

## ***Full Transmission Rebound in October Scenario with Vaccination strategies***

Figure 4a shows that vaccination has a significant impact on mpox incident cases over time, especially during an outbreak scenario. For instance, doubling the likelihood of vaccination can reduce the number of cases in a rebound scenario to levels comparable with the status quo.

Additionally, the effectiveness of targeted vaccination becomes more apparent in rebound scenarios. Distributing the same number of weekly vaccines but specifically targeting PWH can lead to a substantial reduction in new cases. Conversely, focusing vaccination efforts on any single racial group leads to an increase in cases. This may be attributed to the social mixing patterns within partnerships, where targeting one race group could lead to a saturation effect. See Figure 4b.

**Appendix Figure 4**

**REFERENCES**

1. Nguyen, A. *et al.* Are Unequal Policies in Pre-Exposure Prophylaxis Uptake Needed to Improve Equality? An Examination Among Men Who Have Sex with Men in Los Angeles County. *AIDS Patient Care STDs* **36**, 300–312 (2022).

2. U.S. Census Bureau (2022). Los Angeles County, CA - Profile data - Census Reporter. https://censusreporter.org/profiles/05000US06037-los-angeles-county-ca/.

3. Lieb, S. *et al.* Statewide Estimation of Racial/Ethnic Populations of Men Who Have Sex with Men in the U.S. *Public Health Rep.* **126**, 60–72 (2011).

4. Centers for Disease Control and Prevention. Clinical Recognition | Mpox | Poxvirus | CDC. https://www.cdc.gov/poxvirus/mpox/clinicians/clinical-recognition.html (2023).

5. New York Department of Health. *What to Expect After Your Monkeypox Vaccine*. 2 https://www.health.ny.gov/diseases/communicable/zoonoses/mpox/docs/jynneos_what_to_expect.pdf (2022).

6. Adler, H. *et al.* Clinical features and management of human monkeypox: a retrospective observational study in the UK. *Lancet Infect. Dis.* **22**, 1153–1162 (2022).

7. Centers for Disease Control and Prevention. Interim Clinical Considerations for Use of JYNNEOS and ACAM2000 Vaccines during the 2022 U.S. Mpox Outbreak | Mpox | Poxvirus | CDC. https://www.cdc.gov/poxvirus/mpox/clinicians/vaccines/vaccine-considerations.html (2023).

8. Moore, M. J., Rathish, B. & Zahra, F. Mpox (Monkeypox). in *StatPearls* (StatPearls Publishing, Treasure Island (FL), 2023).

9. Vaughan, A. M. *et al.* A large multi-country outbreak of monkeypox across 41 countries in the WHO European Region, 7 March to 23 August 2022. *Eurosurveillance* **27**, 2200620 (2022).

10. Mitjà, O. *et al.* Monkeypox. *Lancet Lond. Engl.* **401**, 60–74 (2023).

11. Palich, R. *et al.* Viral loads in clinical samples of men with monkeypox virus infection: a French case series. *Lancet Infect. Dis.* **23**, 74–80 (2023).

12. Antinori, A. *et al.* Epidemiological, clinical and virological characteristics of four cases of monkeypox support transmission through sexual contact, Italy, May 2022. *Eurosurveillance* **27**, 2200421 (2022).
